# Supplementary material for: Chromothripsis during telomere crisis is independent of NHEJ, and consistent with a replicative origin
Source: Genome Res. 2019 May;29(5):737–49. doi: 10.1101/gr.240705.118 (PMC6499312; doi:10.1101/gr.240705.118)
Supplement: Supplemental Material [file supp_gr.240705.118_Supplemental_file_1.zip › contigs/annotated_contigs/DB48/contig.2.DB48_length_499_mean_cov_5.19038076152.docx]

**DB48_length_499_mean_cov_5.19038076152**

```
ATATATCATAACACTTAGTCACCCTTTAGAATTTAATACAAAACAGATGCCGAAAGAGAAATTGCAAAATGAAGGTAGAATTTTAAAGA
 >chr11:100075790-100076018 - E=1e-125
TGTCTTAGAAATAATGGCCTCTCAAAAACCCAACTCTTTGAAAATCACTTTGAGAAATAACATAGGAGTCAATCTAAAAACACAATACT

TACATATCAATAGTATTTCAAAATGTAAAACTGAACTGTTTGCT|TATATA|TATATATATATATTAGTCAATGTTTGTTTCTTGGTGA
                                             >chr11:100074802-100075048 - E=3e-129
GCTACTCTAAATGGCTAGTTTTTAGTTCATATACTCCTATGTCTACTGATTCCTTTAATGACTACAAATATTTATTTGGTCTTTACTGT

GCACCAGGTACTGTGTTAAGCCCAGCAAATAAGTATGTTCCTAACTAACTTTCAGAAACATAAAGACTAGAAGAGGAAAAAAAAACTGC

GCAAATGTTTAAAGCAGCATAGATCC|CCGAGGCGGGACCTGCCAGGCCTCACGGGC
```
